# Botox, Abdominal Wall Transection, and Body Positioning: A Case of Complex Abdominal Wall Reconstruction With Seat Belt Syndrome

**DOI:** 10.7759/cureus.19043

**Published:** 2021-10-25

**Authors:** William T Head, Christopher S Thomas, Evert Eriksson

**Affiliations:** 1 Department of Surgery, The Medical University of South Carolina, Charleston, USA

**Keywords:** abdomen ventral hernia, seat belt sign, mesh repair, botox injections, abdominal wall reconstruction, seat belt syndrome

## Abstract

Seat belt syndrome (SBS) represents all injury profiles associated with seat belt injuries and motor vehicle crashes (MVCs). Seat belt syndrome classically presents with a superficial seat belt sign that may signify deeper intra-abdominal and/or spinal involvement. The amount of force transmitted from the restraint to the passenger ultimately dictates the amount and severity of the injury. We present a unique case of a 59-year-old female involved in a motor vehicle crash with multiple traumatic injuries, including seat belt syndrome, abdominal wall transection, and bowel injuries. She later had reconstruction of her traumatic abdominal wall hernias (TAWHs). Three unique approaches were used in the management of her traumatic abdominal wall hernias: (1) preoperative Botulinum toxin (Botox) injections, (2) operative use of biologic and bioabsorbable meshes in contaminated fields, and (3) postoperative physical therapy and body positioning. The patient did not experience any recurrence of these hernias after her abdominal wall reconstruction and remains alive at the time this case was written. The diagnostic criteria and surgical management of traumatic abdominal wall hernias have yet to be established, and the case presented here provides approaches that should serve as future areas for study.

## Introduction

More than six million motor vehicle crashes (MVCs) occur annually in the United States, resulting in more than two million emergency department visits [1,2]. MVCs result in over 35,000 annual deaths and are therefore the leading cause of death before age 30 [1]. Seat belts have effectively reduced the mortality associated with MVCs by an estimated 45%-75% [3]. Nevertheless, seat belt-associated injuries can occur due to the blunt force trauma transmitted between the restraint and the body on impact. In particular, the traditional three-point harness is designed to transmit force to the clavicle, chest wall, and superior pelvis [4]. The excess force during MVCs commonly results in superficial and deep injuries along these areas. Superficially, the appearance of abrasions or contusions in the distribution of the seat belt across the chest and abdomen is referred to as a “seat belt sign” [5]. A seat belt sign may signify deeper injuries of greater concern often involving the bowel, mesentery, solid organ, chest wall, and/or lumbar spine. In patients who survive an MVC, the presence of a seat belt sign has been associated with increased incidence of hollow viscus injury by eight times, solid organ by 5.7 times, and ribs by 2.4 times relative to those without a seat belt sign [6]. Altogether, the appearance of a seat belt sign with intra-abdominal and/or spinal injuries is referred to as “seat belt syndrome” (SBS) [7].

## Case presentation

**Figure 1:**
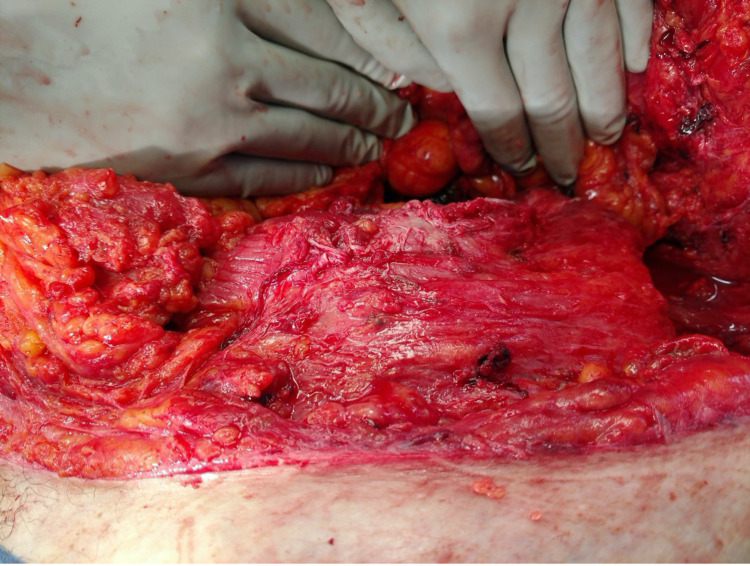
Left Pelvic Brim at the Initial Exploratory Laparotomy

**Figure 2:**
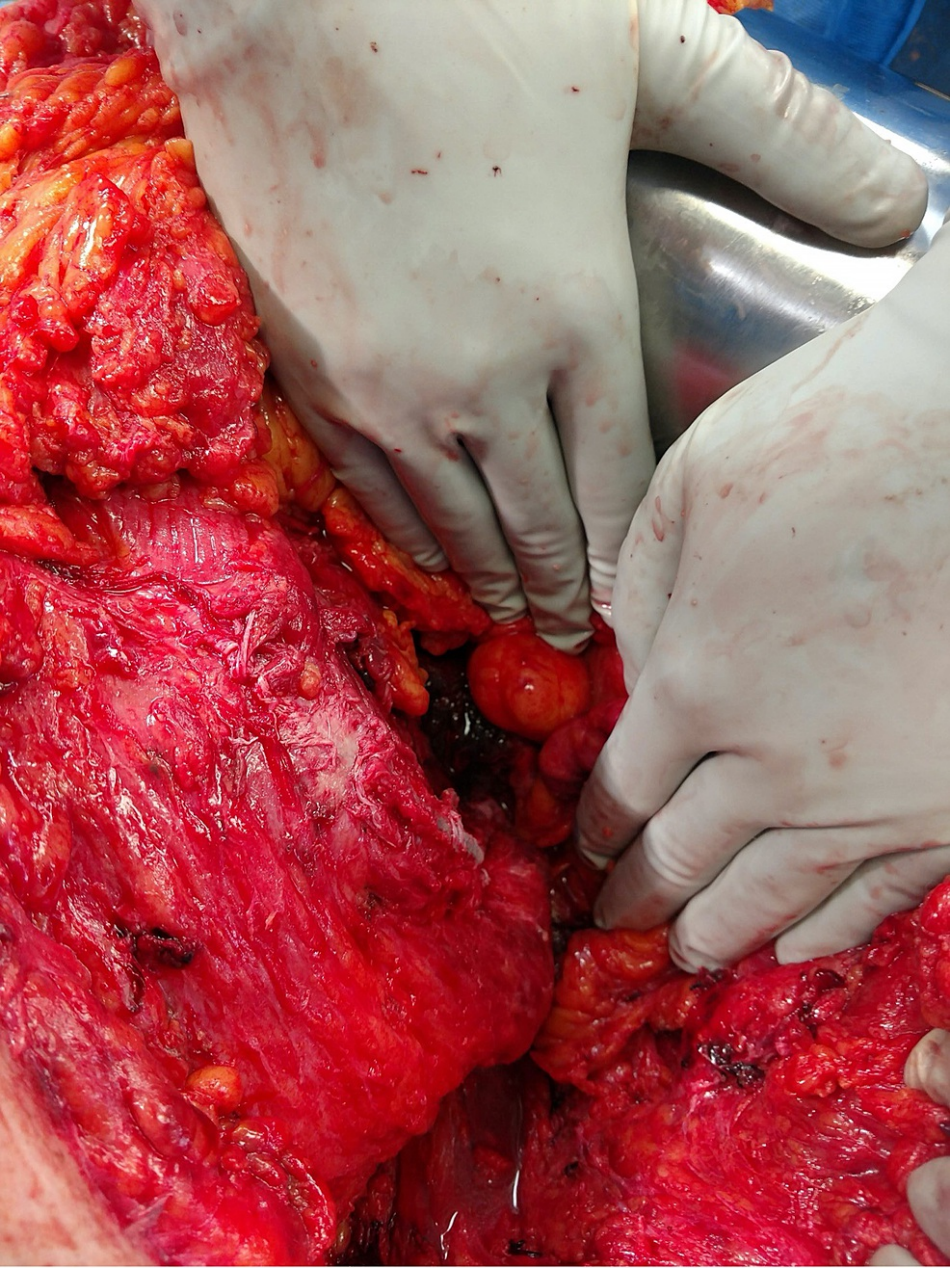
Avulsion of the Lateral Abdominal Wall off the Left Pelvic Brim

We present the case of a 59-year-old female who presented to our academic level I trauma center with SBS secondary to an MVC. The initial workup identified the following injuries: eviscerated bowel, degloving of the lower abdominal wall, bilateral groin lacerations, left common iliac artery dissection, retroperitoneal hematoma, left femoral nerve injury, transected musculature (psoas, left obliques, and bilateral rectus abdominis), and various fractures (seventh cervical vertebra, left transverse process of the first lumbar vertebra, anterior superior endplate of the second lumbar vertebra, left 10th rib, right third proximal phalanx, right ankle, and nasal bones). She was taken to the operating room (OR) for an emergent exploratory laparotomy. Figures 1 and 2 display some of the intraoperative findings during the initial assessment.

The abdominal wall had been sheared from the left anterior superior iliac spine to the paraspinous muscles posteriorly. A systematic evaluation of the abdomen revealed areas of devitalized small bowel mesentery and sigmoid colon transection that both required segmental resections and were left in discontinuity during the initial operation.

**Figure 3:**
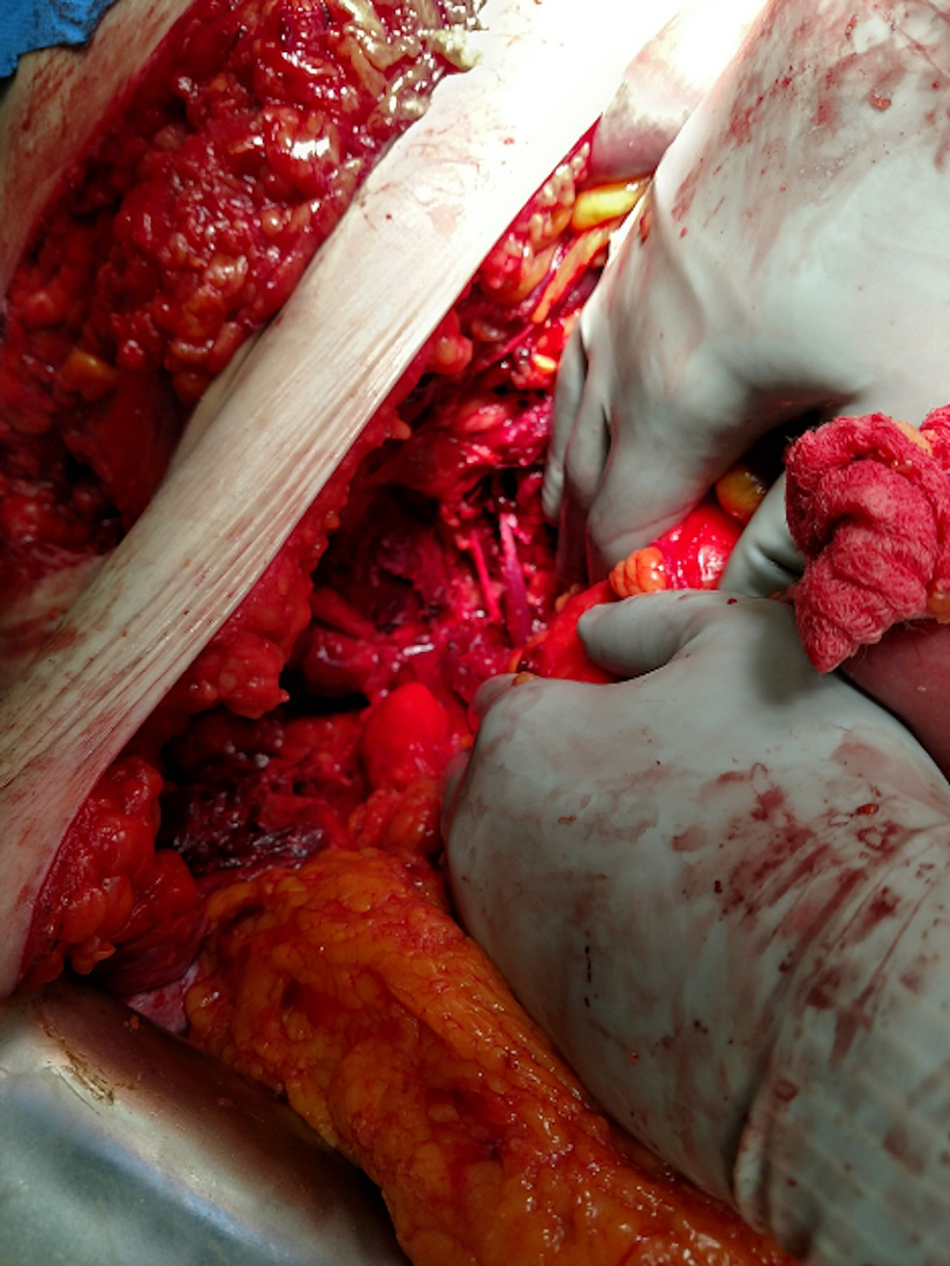
Left Femoral Nerve and Partially Transected Psoas Muscle

She was taken back to the OR on hospital day (HD) 1 for further small bowel and sigmoid colon resection and anastomosis. Evaluation of the abdominal wall revealed transected parts of the right internal and external abdominal obliques, transversalis abdominis, and rectus abdominis musculature with partial transection of the psoas muscle. The transection extended circumferentially along the left side to the paraspinous muscles. The femoral nerve was exposed with additional transection of the ilioinguinal nerve and other cutaneous nerves of the retroperitoneum (Figure 3).

Then, 24 x 13 cm of devitalized skin, fat, and muscles was debrided. The patient was closed again with a negative pressure device with an intent to return shortly for further abdominal wall closure.

**Figure 4:**
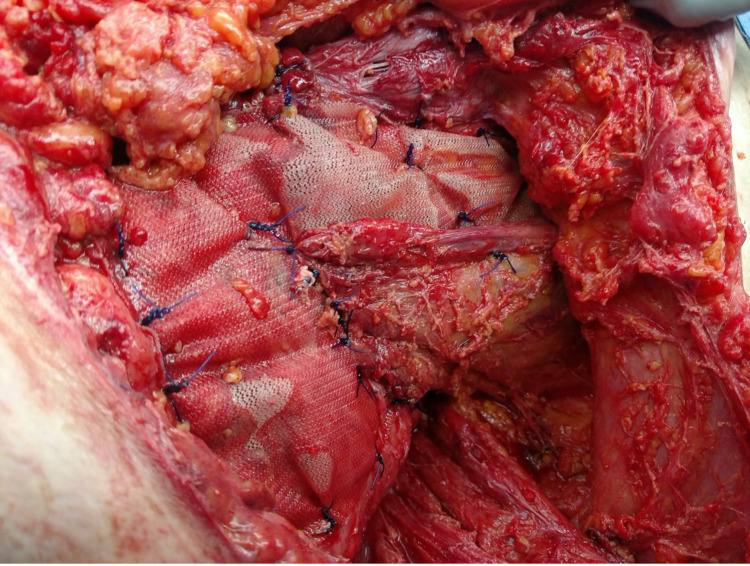
Bioabsorbable Mesh Placement Over Internal Oblique and Pelvic Brim With External Oblique Muscle Advanced Over the Mesh

On HD 3, the patient returned to the OR for repair of the transected left abdominal wall. The posterior abdominal wall was sutured together, and the internal oblique and transversalis abdominis musculature were sutured to the fascial insertions at the pelvic brim. The external abdominal oblique musculature was elevated off the internal oblique to provide length and bring it down to the anterior abdominal wall. An absorbable poly-4-hydroxybutyrate (P4HB) mesh was placed over the fascia lata across the pelvic brim and onto the internal oblique fascia, suturing it to the fascia lata and internal oblique musculature (Figure 4).

The external oblique was then brought over the mesh and sutured down with transfascial sutures through the abdominal wall, including both the mesh and the external oblique. Due to third spacing and edema, the midline wound was unable to be closed. The rectus sheath also had considerable tissue loss and could not be fully reapproximated over the left rectus abdominis. A negative pressure device was again placed over the abdomen with plans to return shortly for an underlay repair.

**Figure 5:**
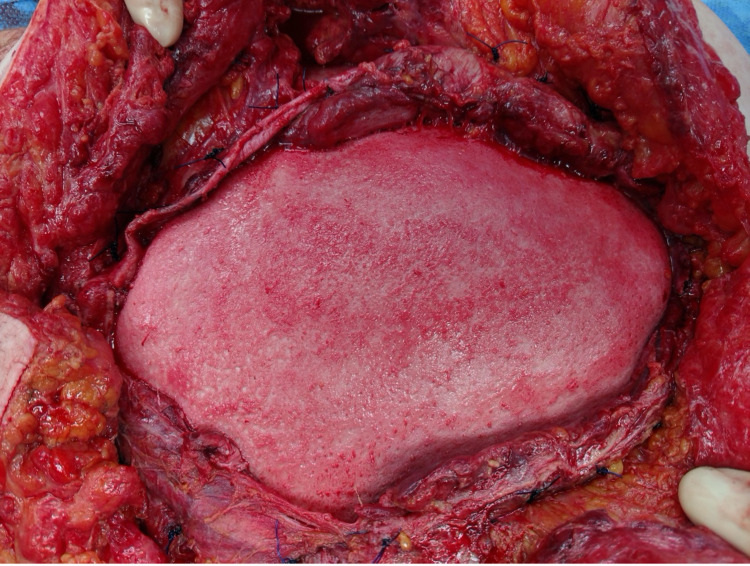
Biologic Mesh Covering the Exposed Bowel as a Bridge for Planned Ventral Hernia Formation and Future Repair

On HD 5, the patient returned to the OR. The midline fascia could not be reapproximated to its original position due to edema, so a cadaveric dermis mesh underlay was performed with additional repair of an incarcerated ventral hernia (Figure 5).

**Figure 6:**
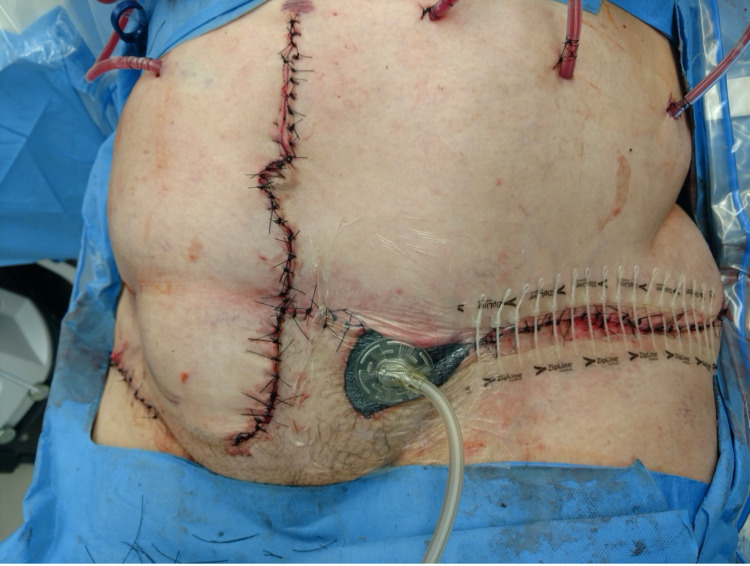
Use of Wound Vac and Zip Line to Granulate the Small Area Unable to be Closed and to Offload the Tight Suture Closure of the Lateral Abdominal Wall Due to Skin Loss

Granulating mesh was chosen since a large area in the left lower quadrant could not be covered by tissues without transfer. A negative pressure device was again placed. On HD 7, the patient returned to the OR for washout and possible abdominal closure; however, the skin and soft tissue were unable to be brought together primarily, and 100 cm^2^ of devitalized skin, fat, and muscle was debrided. On HD 9, the patient returned to the OR for fasciocutaneous advancement flap on the left to close the soft tissue gap. Multiple drains were placed to prevent postoperative seroma collection. Scarpa's fascia and the overlying skin were advanced to provide adequate skin coverage of the left abdomen. This layer was sutured to the anterior abdominal fascia and reapproximated over the entire 63 cm length of the incisions to close these spaces. All the skin laceration incisions were able to be reapproximated except for a 4 x 6 cm location overlying the mons pubis. A negative pressure device was placed here. A zip line closure was also placed over the left abdominal closure to help reapproximate the skin and offload the incision tension (Figure 6).

The patient was maintained in 30 degrees of flexion with physical therapy (PT) support for one week postoperatively to limit abdominal wall tension. She was advanced to a pivot to chair without extension on HD 19. The wound vac over the mons pubis was removed on HD 23. After she became tachycardic on HD 26, imaging revealed an anterior abdominal wall fluid collection. Interventional radiology (IR) performed a percutaneous drainage catheter placement into the collection. Cultures revealed methicillin-resistant *Staphylococcus aureus*, and she was started on IV vancomycin. Another IR drain was placed into a right anterior abdominal wall collection on HD 30. She improved incrementally and was discharged on HD 53.

**Figure 7:**
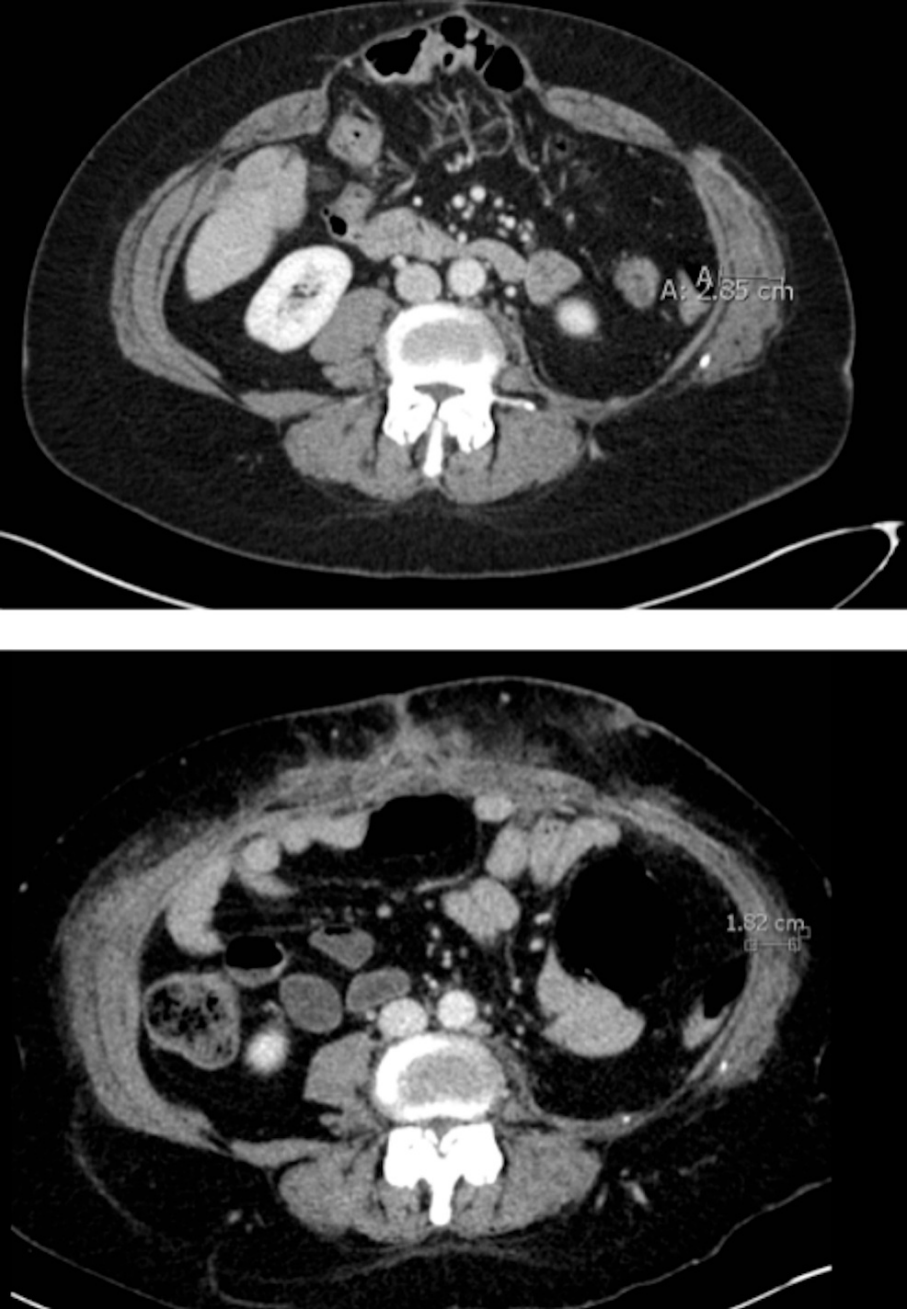
Pre- and Post-Botox Injection CT Scans These CT scans of a cross-section of the abdomen show how the Botox enabled us to pull her abdominal muscles back into position. In the top image, the hernia is visible before surgical intervention. Measurement shows that the right abdominal muscle has retracted and consequently has thickened to a width of 2.85 cm. In the lower image, the muscle has been stretched thinner, so the new measured width is 1.82 cm, and the muscle was pulled back to the center of her body.

The patient remained stable for approximately one year and returned for outpatient evaluation of an incarcerated ventral hernia 366 days after the initial admission. Given that she had already undergone component separation and complex abdominal wall reconstruction on the left side from the initial injury, a novel approach was taken with Botulinum toxin (Botox) to increase the likelihood of primary fascial closure. She received 300 total units of Botox diluted 3:1 with sterile saline (50 each in the left external intercostals, right external intercostals, left internal intercostals, right internal intercostals, left transversus abdominis, and right transversus abdominis) 418 days after the initial hospital admission. The patient was readmitted for open ventral hernia repair with mesh and a myofascial advancement flap on the right 433 days after the initial admission. The Botox injections had allowed for the fascia to be approximated to the midline (Figure 7).

A mesh with a nonabsorbable polypropylene layer surrounded by polydioxanone reinforced the repair. The patient was last seen 541 days after the initial accident, recovering well with no signs of hernia or reconstruction failure.

## Discussion

Approximately 9% of all blunt trauma patients have abdominal wall injuries with less than 1.5% demonstrating traumatic abdominal wall hernias (TAWHs) [8]. SBS has rarely been identified as a cause of TAWH with its associated musculoskeletal and viscus injuries [9]. TAWHs ultimately result from blunt trauma disrupting the abdominal wall musculature and fascia. The diagnostic criteria and, more importantly, appropriate management of TAWH have yet to be well established [10]. The case presented here identifies valuable approaches to complex abdominal wall reconstruction for TAWH with three particular areas of interest: (1) preoperative Botox injections, (2) operative use of mesh versus primary repair in contaminated fields, and (3) postoperative PT and patient positioning. Botox has recently been identified as a minimally invasive adjunct to increase tissue mobility and facilitate primary fascial closure [11,12]. The preoperative administration of Botox in the present case enabled for closer fascial approximation, limiting the amount of mesh required for ventral hernia repair. No major complications have been associated with Botox in ventral hernia repair, and the patient presented here did not develop any postoperative complications from Botox injection [11].

The operative approach to TAWH often varies in terms of mesh versus primary closure and acute versus delayed repair. The relative benefits of mesh include repairing defects too large for primary closure and less chance for recurrence; however, mesh has historically been reported as an absolute contraindication in peritoneal contamination with higher infection rates [9,13,14]. Some have begun to question this contraindication and instead now advocate for the use of biologic meshes as safe alternatives in the trauma setting [10,15]. Recent publications have even suggested that synthetic meshes may provide similar outcomes in both contaminated and clean repairs [16]. Studies assessing the optimal postoperative positioning and advancement for complex abdominal wall reconstruction patients have yet to be published, yet it is commonly used by plastic surgeons during abdominoplasty [17]. 

The case presented here included an initial damage control laparotomy, followed by delayed repair with bioabsorbable and biologic meshes four and six days after the initial injury. In particular, the reconstruction of the left abdominal wall four days after the initial injury was performed by suturing the internal oblique and transversalis fascia down to the fascia lata at the level of the pelvic brim. The area was reinforced with a P4HB mesh, which was sutured as an on-lay mesh over the internal oblique and fascia lata. The external oblique was then brought over the mesh and sutured into position, resulting in a mesh inlay between the internal and external oblique muscle layers. P4HB was selected because it could be placed in a contaminated field and absorbed over time. It also allows for an orderly arrangement of mature, type I collagen with minimal inflammation in the surrounding tissues [18]. The bridging cadaveric dermis mesh closure was a subsequent planned ventral hernia repair six days after the initial injury. The authors' experience has suggested that cadaveric mesh offers improved granulation relative to other available products. Infection, skin flap viability, and skin integrity were primary concerns at the time of repair, and the cadaveric mesh provided an ideal solution in this scenario.

A synthetic mesh was then used 433 days after the initial injury with the Botox injections approach described earlier. A fasciocutaneous advancement flap was performed 10 days after injury to close a soft tissue gap in the left abdomen. This repair was also supported using a bioabsorbable mesh in an inlay fashion between the internal and external obliques. Postoperative body positioning techniques and PT were advanced over the course of several weeks to offset the tension of the closure and the tension of the muscle and skin. In particular, the patient was started at a 30-degree flexed position with progressive advancement later on. The patient presented here ultimately progressed well with no major complications from her positioning therapy.

## Conclusions

Overall, the case presented here is rare and provides a unique approach to the management of SBS and TAWH. The use of novel strategies, including preoperative Botox injections, operative use of biologic and bioabsorbable meshes in previously contaminated fields, and postoperative body positioning, may prove to be of use moving forward. While the diagnostic criteria and management of TAWH have yet to be defined, future studies should assess the specific presentation of TAWH in SBS and the validity of the management approaches presented here. Moving forward, the practicing trauma surgeon should be aware of these strategies to manage various forms of complex abdominal wall reconstructions.
